# Global DNA methylation and cognitive and behavioral outcomes at 4 years of age: A cross‐sectional study

**DOI:** 10.1002/brb3.1579

**Published:** 2020-02-28

**Authors:** Rachael M. Taylor, Roger Smith, Clare E. Collins, David Mossman, Michelle W. Wong‐Brown, Eng‐Cheng Chan, Tiffany‐Jane Evans, John R. Attia, Nick Buckley, Karen Drysdale, Tenele Smith, Trent Butler, Alexis J. Hure

**Affiliations:** ^1^ Priority Research Centre for Reproductive Science University of Newcastle Callaghan NSW Australia; ^2^ Faculty of Health and Medicine School of Medicine and Public Health University of Newcastle Callaghan NSW Australia; ^3^ Hunter Medical Research Institute New Lambton Heights NSW Australia; ^4^ Faculty of Health and Medicine School of Health Sciences University of Newcastle Callaghan NSW Australia; ^5^ Priority Research Centre in Physical Activity and Nutrition University of Newcastle Callaghan NSW Australia; ^6^ Department of Molecular Medicine NSW Health Pathology John Hunter Hospital New Lambton Heights NSW Australia; ^7^ Faculty of Health School of Biomedical Sciences and Pharmacy University of Newcastle Callaghan NSW Australia; ^8^ Clinical Research Design IT and Statistical Support (CReDITSS) Unit Hunter Medical Research Institute New Lambton Heights NSW Australia; ^9^ School of Psychology and Exercise Science Murdoch University Murdoch WA Australia; ^10^ Faculty of Science School Psychology University of Newcastle Callaghan NSW Australia; ^11^ Priority Research Centre for Generational, Health and Ageing University of Newcastle Callaghan NSW Australia

**Keywords:** behavior, cognition, global DNA methylation, nutrition, one‐carbon metabolism, postnatal

## Abstract

**Background:**

Accumulating evidence suggests that breastfeeding exclusivity and duration are positively associated with child cognition. This study investigated whether DNA methylation, an epigenetic mechanism modified by nutrient intake, may contribute to the link between breastfeeding and child cognition. The aim was to quantify the relationship between global DNA methylation and cognition and behavior at 4 years of age.

**Methods:**

Child behavior and cognition were measured at age 4 years using the Wechsler Preschool and Primary Scale of Intelligence, third version (WPPSI‐III), and Child Behavior Checklist (CBC). Global DNA methylation (%5‐methylcytosines (%5mC)) was measured in buccal cells at age 4 years, using an enzyme‐linked immunosorbent assay (ELISA) commercial kit. Linear regression models were used to quantify the statistical relationships.

**Results:**

Data were collected from 73 children recruited from the Women and Their Children's Health (WATCH) study. No statistically significant associations were found between global DNA methylation levels and child cognition or behavior (*p* > .05), though the estimates of effect were consistently negative. Global DNA methylation levels in males were significantly higher than in females (median %5mC: 1.82 vs. 1.03, males and females, respectively, (*p* < .05)).

**Conclusion:**

No association was found between global DNA methylation and child cognition and behavior; however given the small sample, this study should be pooled with other cohorts in future meta‐analyses.

## Graphical abstract

This study quantified the relationship between global DNA methylation and cognition and behavior at 4 years of age. Data were collected from 73 children indicated no statistically significant associations were found between global DNA methylation levels and child cognition or behavior (*p* > .05). Global DNA methylation levels in males were significantly higher than in females (median %5mC: 1.82 vs. 1.03, males and females, respectively, (*p* < .05)).

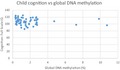

## 1 BACKGROUND

The Developmental Origins of Health and Disease (DOHaD) hypothesis postulates that environmental exposures in utero or during the postnatal period may alter physiological programming and disease risk in adulthood (Gluckman, Hanson, and Pinal (2005)). Early‐life exposures, including maternal smoking (Polanska et al., 2017; Moylan et al., 2015), low birthweight (Arcangeli, Thilaganathan, Hooper, Khan, & Bhide, 2012; Miller, DeBoer, & Scharf, 2017), and inadequate nutrition (Darling et al., 2017; Liu and Raine, 2017), have been associated with behavioral and cognitive deficits in later life. Epigenetics is postulated to be a possible mechanism involved in the association between early‐life exposures and human diseases (Bauer et al., 2016; Radtke et al., 2011; Tobi et al., 2015). Epigenetic processes, including DNA methylation, histone modifications, and noncoding RNAs, are involved in brain development, including neuronal proliferation and differentiation, which provides neural plasticity in response to environmental exposures (Stadler et al., 2005; Wang et al., 2012).

The nutritional environment during prenatal and postnatal development can influence the establishment and maintenance of DNA methylation patterns of the genome (Cooney, Dave, & Wolff, 2002; Dominguez‐Salas et al., 2014; Kotsopoulos, Sohn, & Kim, 2008; Obermann‐Borst et al., 2013). Dietary nutrients, including methionine, choline, betaine, folate, choline, and vitamins B2, B6, and B12, are involved in the biochemical pathways of one‐carbon metabolism (Van den Veyver, 2002). These nutrients act as methyl donors and cofactors in a series of biochemical reactions that regulate the production of the universal methyl donor, S‐adenosylmethionine (SAM). DNA methylation involves the transfer of a methyl group (CH_3_) from SAM to a DNA cytosine–guanine nucleotide pair bound by phosphate (CpG; Law & Jacobsen, 2010). To establish new methylation patterns, methyl groups are transferred to CpG nucleotides by DNA methyltransferase (DNMT) enzymes, DNMT3a and DNMT3b @@(Okano, Bell, Haber, & Li, 1999), while, during DNA replication, DNMT1 copies the DNA methylation patterns from the parental DNA strand to the synthesized daughter strand (Li, Bestor, & Jaenisch, 1992). The transcription of genes are inhibited by methylated DNA bound to methyl‐CpG binding proteins (MBPs) which triggers the recruitment of chromatin remodeling proteins to form compact inactive chromatin, thereby preventing transcription factors from binding (Bird, 2002; Klose & Bird, 2006). Gene expression and physiological function can be altered if DNA methylation occurs within the promoter regions (Jones & Taylor, 1980).

During human development, epigenetic programming, including DNA methylation, is essential for establishing cell‐ and tissue‐specific transcriptional regulation (Wolffe & Matzke, 1999). For example, upon human fertilization, genome‐wide demethylation occurs, while the prenatal and postnatal periods are characterized by significant remethylation to acquire tissue‐specific functionality (Numata et al., 2012; Spiers et al., 2015). Emerging evidence suggests that during prenatal brain development, significant DNA methylation occurs during neurulation, which coincides with the differentiation of the neuroprogenitor cells (Chen, Damayanti, Irudayaraj, Dunn, & Zhou, 2014). During prenatal and postnatal life, significant epigenetic reprogramming appears to occur during the initiation of neurodevelopmental processes, including neural differentiation and synaptogenesis (Chen et al., 2014; Lister et al., 2013). Evidence from animal models suggests that DNA methylation is involved in the development of cognitive abilities and behavior via the cellular regulation of synaptogenesis and synaptic pruning (Feng et al., 2010; Gapp et al., 2016; Meadows et al., 2015; Halder et al., 2016).

Epidemiological studies suggest that child cognitive development is related to early‐life nutrition. A large (*n = *13,889) cluster‐randomized control trial demonstrated that cognitive development at 6.5 years was strongly associated with the duration of exclusive (≥ 3 months) breastfeeding, after adjustments for potential confounders of geographical location, age at follow‐up, sex, birthweight, and both maternal and paternal education (Kramer et al., 2008). These findings were also confirmed in a systematic review and meta‐analysis of 17 studies demonstrating a positive association between breastfeeding and child cognition (up to the age of 15 years), after adjusting for maternal IQ (Horta, 2015). Furthermore, the Women and Their Children's Health (WATCH) study, a small but detailed prospective longitudinal birth cohort, previously reported significant differences in serum nutrients required for one‐carbon metabolism and DNA methylation between breastfed infants compared to formula‐fed infants (Hure, Collins, & Smith, 2012). More specifically, breastfed infants had significantly lower plasma B12 and folate, and higher homocysteine levels compared to formula‐fed infants at 6 month of age (Hure, Collins, & Smith, 2012). Therefore, it may be hypothesized that lower B12 and folate levels may restrict the conversion of methionine to S‐adenosylmethionine subsequently reducing DNA methylation and changing the regulation of the epigenome. Furthermore, Cheatham and Sheppard (2015) demonstrated that higher choline and lutein levels in breastmilk were associated with higher memory recognition in infants (*n = *55) at 6 month, suggesting that adequate postnatal nutrition is important for establishing specific DNA methylation patterns that support brain development and cognitive function.

In the Growing Up Singapore Towards Healthy Outcomes (GUSTO) cohort, greater cord blood DNA methylation was associated with lower externalizing behavior scores in one‐year‐old children (*n = *108; Lillycrop et al., 2015). The UK Southampton Women's Survey (SWS) showed greater cord blood DNA methylation was associated with higher intelligence quotient (IQ) scores at 4 years of age (*n = *175) and higher executive memory function at 7 years of age (*n = *200; Lillycrop et al., 2015). In the US Rhode Island Child Health Study (RICHS) cohort, placental DNA methylation was positively associated with attention in newborns (*n = *335; Lesseur et al., 2014). The US Conditions Affecting Neurocognitive Development and Learning in Early Childhood (CANDLE) cohort found no association between genome‐wide DNA methylation of cord blood and cognition outcomes in children at 1 year of age (*n = *168; Krushkal et al., 2014). These studies have reported both positive and negative associations between DNA methylation patterns and child cognition or behavior; therefore, further evidence is required from comparable studies before more definitive conclusions can be drawn.

We hypothesized that the association between breastfeeding and child cognition may be attributed to epigenetic mechanisms. Therefore, the present study aimed to test whether buccal DNA methylation at age four is associated with cognition and behavior outcomes in a population of Australian children.

## 2 METHODS

### 2.1 Study population

This study included data from mothers and their children enrolled in the WATCH cohort (Hure, Collins, Giles, Wright, & Smith, 2012). The following methods have been described previously (Taylor et al., 2018); briefly, pregnant women were recruited from the antenatal clinic at the John Hunter Hospital (JHH), New South Wales (NSW), Australia, from July 2006 to December 2008. All women who were less than 18 weeks pregnant, lived in the local area, and were able to attend JHH were eligible to participate. Women were recruited by midwives, local media coverage, or word of mouth. A consent rate of 61% was achieved for pregnant women who were approached to participate in the study, and 181 women were enrolled in the study. The mothers and their children attended regular follow‐up visits during pregnancy (19, 24, 30, and 36 weeks of gestation) and the postnatal period (three‐monthly intervals during the first year after birth and then annually until age 4 years). The WATCH study received ethics approval from the Hunter New England Research Ethics Committee (06/05/24/5.06), and all participants gave written informed consent.

### 2.2 DNA collection

Child buccal cheek swabs were collected from the WATCH cohort at 4 years of age for DNA extraction, using the Isohelix buccal DNA isolation kits (Isohelix, Cat. no. SK‐1S), as previously described (Taylor et al., 2018). In preparation for the sample collection, children refrained from eating and drinking for 45 min prior to their study visit. To collect the buccal cells, a research assistant firmly rubbed a sterile swab head against the child's inside cheeks for approximately 20 s on both sides. The samples were stored in a sterile 5‐ml tube at −80°C. Buccal cheek cell samples were chosen to examine DNA methylation because they are the least invasive sample to collect, which is appropriate for a cohort of young children, and the cell population is more homogenous compared to blood samples (Lowe et al., 2013). Compared with blood, buccal samples have also shown greater correlation in the hypomethylated tissue‐specific differentially methylated regions (tDMRs) with hypomethylated regions in other tissues (brain, full‐term placenta, liver, kidneys, pancreas, skeletal muscle, and sperm; Lowe et al., 2013).

### 2.3 DNA extraction

DNA samples were extracted from the buccal cells using the Qiagen Gentra Puregene Buccal Cell Kit (Qiagen, Cat no. 158845), as previously described (Taylor et al., 2018). The swab heads were removed from the handle and added to 2‐ml tubes containing 300 µl of cell lysis solution and 1.5 µl proteinase K. After incubating at 55°C overnight, the swab collection heads were discarded and 1.5 µl RNase A solution was added to the tubes. The DNA samples were incubated at 37°C for 1 hr prior to adding 100 µl protein precipitation solution to the tubes. Precipitated proteins and insoluble cellular debris were pelleted by centrifuging the 2‐mL tubes at 13,000 *g* for 3 min and incubating them on ice for 5 min. This step was repeated to form a tight protein pellet. The supernatant was collected into sterile 1.5‐ml tubes, and 300 µl of isopropanol and 0.5 µl of glycogen solution were added to the precipitated DNA. The tubes were gently inverted and centrifuged at 13,000 *g* for 5 min. The supernatant was discarded, and the DNA pellets were resuspended in 300 µl of ethanol solution (70%) and centrifuged at 13,000 *g* for 1 min. The supernatant was discarded, and the DNA pellets were left to dry at room temperature for 5 min. The DNA pellets were resuspended in 20 µl of DNA hydration solution and centrifuged for 3 min at 13,000 *g*. The DNA samples were incubated at room temperature overnight. The DNA concentration of each sample was estimated using a NanoDrop 1000 (Thermo Fisher Scientific).

### 2.4 Quantification of genome‐wide DNA methylation

Genome‐wide DNA methylation of child buccal cheek swabs was analyzed using an (indirect) ELISA‐based commercial kit (MethylFlash Methylated DNA 5‐mC Quantification Kit (Colorimetric), EpiGentek Group Inc., Cat. no. P‐1034‐96), as previously described (Taylor et al., 2018). Briefly, 0.4–5 µl of sample DNA (25–100 ng input DNA) was bound to strip wells with a high DNA affinity. Methylated DNA was detected using capture and detection antibodies to 5‐methylcytosine (5‐mC) and then quantified colorimetrically by reading the absorbance at 450 nm, using a SPECTROstar Nano plate reader (BMG Labtech). In this ELISA, the amount of methylated DNA is proportional to the optical density (OD). In human somatic cells, 70%–80% of CpG dinucleotides are methylated, which constitute <1% of the genome (Ehrlich et al., 1982). Therefore, the percentage of detected 5‐mC is expected to be low, due to the low prevalence of CpGs in the human genome. All DNA samples were analyzed in triplicates; however, if the amount of DNA amount was limited (<0.5 ng per 1 ml), the samples were analyzed in duplicates, and mean values were used for the statistical analysis. A standard curve was generated according to the manufacturer's instructions and used to quantify the percentage of methylated DNA in the total DNA sample.

### 2.5 Cognition and behavioral assessment

#### 2.5.1 Cognition

Child cognition was assessed using the Wechsler Preschool and Primary Scale of Intelligence (WPPSI‐III Australian; Wechsler, 2002a) which is suitable for children aged 4–7.3 years (PsychCorp). The cognition assessments were individually administered by a research psychologist at the four‐year study visit. The WPPSI‐III is widely cited for preschool children and has satisfactory criterion validity, correlating with Wechsler Preschool and Primary Scale of Intelligence, revised version (WPPSI‐R), Wechsler Intelligence Scale for Children, third edition (WISC‐III), and Wechsler Intelligence Scale for Children, fourth edition (WISC‐IV; Wechsler, 2002b; Wechsler, 2004). The scale produces three main composite scores: Full‐Scale Intelligence Quotient (FSIQ), Performance Intelligence Quotient (PIQ), and Verbal Intelligence Quotient (VIQ), as well as two additional composite scores: Processing Speed Quotient (PSQ) and General Language Composite (GLC). The raw scores for performance IQ and verbal IQ are based on the number of subtests successfully completed and are converted to standardized scores according to the child's age. The full‐scale IQ is the combined standardized scores derived from both the performance IQ and verbal IQ. All composite scores have a mean of 100 and a standard deviation of 15.

#### 2.5.2 Behavior

Child behavior was assessed using the Child Behavior Checklist (CBC) for children aged 1.5 to 5 years (Achenbach & Rescorla, 2001), which has demonstrated internal accuracy of the scale across 22 countries, including Australia (Rescorla et al., 2011). The behavior assessments were completed by the primary caregiver of the child during their four‐year study visit. The checklist contains 113 behavioral/emotional problem items (questions) in eight syndrome scales. The syndrome scales include anxious/depressed, withdrawn/depressed, somatic complaints, social problems, thought problems, attention problems, rule‐breaking behavior, and aggressive behavior. The first three syndrome scales combined to produce the internalizing problems score (internalizing broadband scale), and the last two syndrome scales produce the externalizing problems score (externalizing broadband scale). The Total Behaviour Problem Scale summarizes the scores obtained across all scale scores. The checklist items are rated by the child's parent on a three‐point scale, “not true” (0 point), “sometimes true” (1 point), and “often true” (2 points). Scores of the scales are interpreted as normal, borderline, or clinical behavior.

### 2.6 Participant characteristics

Sociodemographic, maternal, and medical information was collected from the WATCH mothers during their first study visit, which has been previously reported (Hure, Collins, Giles, et al., 2012; Taylor et al., 2018).

### 2.7 Statistical analysis

Sample demographic characteristics were summarized as mean and standard deviation for continuous variables and as frequency and percentage for categorical variables. To determine the association of global DNA methylation on cognition and behavior outcomes, robust linear regression model was used. DNA methylation was transformed (natural logarithm) to achieve linearity of the relationship. The association is reported as the expected change in cognition and behavior outcome score per unit increase in the natural logarithm‐transformed global DNA methylation percentage. Each linear regression was adjusted for age as it is associated with both the study predictor (global DNA methylation; Alisch et al., 2012; Teschendorff et al., 2010) and outcomes (cognition and behavior; Harada, Natelson Love, & Triebel, 2013; Murman, 2015). The variable sex is strongly associated with global DNA methylation (Zhang et al., 2011), but there is no strong evidence to suggest that child cognition outcomes, measured by WPPSI‐III at 4 years of age, systematically vary by sex. Therefore, each linear regression was not adjusted for sex. All tests assumed a 5% significance level. All statistical analyses were performed using Statistical Analysis System (SAS) software (version 9.4; SAS Institute).

## 3 RESULTS

Seventy‐three children from the WATCH cohort provided buccal DNA samples and completed cognitive and behavioral testing (41 female and 32 male). Global DNA methylation could not be analyzed in four samples, and six samples could only be analyzed in duplicates rather than triplicates due to limited (<0.5 ng per 1 ml) DNA available. The age of the children at the four‐year follow‐up ranged from 45 to 57 months. The scheduled visits for collecting DNA samples and assessing cognition and behavior was age‐adjusted for children born preterm (<37 completed weeks' gestation, *n = *6).

The characteristics of the WATCH mothers in the study subset are summarized in Table 1. In summary, the mothers within the cohort tended to be highly educated (completed a university degree) and married. The maternal age of the mothers ranged from 18 to 41 years, and most were nonsmokers and had 1–2 live births including the child participating in the WATCH study. The birthweights of the children ranged from 1960 to 5,080 g.

**Table 1:** Characteristics of the WATCH mother–child pairs included in the analysis (*n* = 73)

Characteristics		
Mother	Median (*SD*)	Range
Maternal age (year)	30 (6)	18–41

	*n*	%
Education		
No formal qualification	1	1.4
Year 10 or equivalent	13	18
Year 12 or equivalent	13	18
Trade/apprenticeship	3	4.2
Certificate/diploma	12	17
University degree	23	32
Higher university degree	6	8.5
Missing	2	
Income (per week)		
No income	7	9.7
$1–299	26	36
300–699	25	35
700–999	11	15
Unsure	3	4.2
Missing	1	
Marital status		
Never married	23	32
Married	46	64
Divorced	2	2.8
Widowed	1	1.4
Missing	1	
Maternal smoking		
Yes	7	10
No	62	90
Missing	4	
Live births (>37 weeks of gestation)		
None	1	1.6
1–2	46	75
3–4	14	23.1
>5	1	1.6
Missing	11	
Live preterm births (≤36 weeks of gestation)		
None	39	53.4
1	6	8.2
2	1	1.4
Missing	27	

Child	Mean (*SD*)	Range
Birthweight (g)	3,526 (552)	1,960–5,080
Birth length (cm)	51.4 (2.4)	48–57.5
Head circumference (cm)	34.7 (1.5)	31–39.5

Gender	*n*	%
Male	32	44
Female	41	56

**Figure 1:**
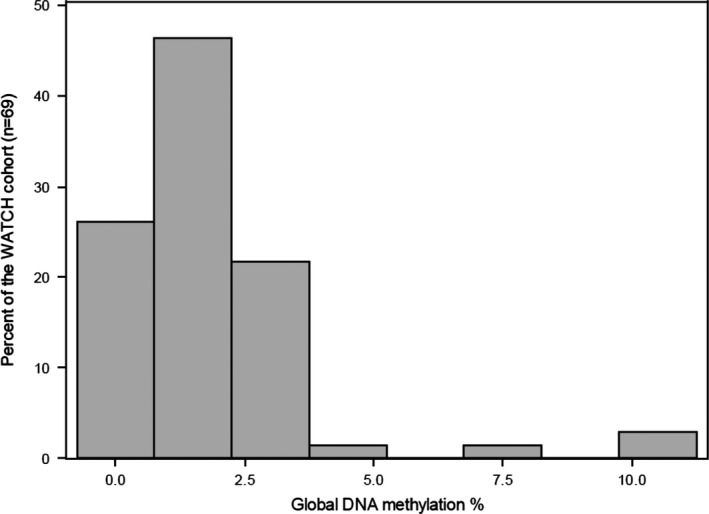
Frequency histograms for the range of global DNA methylation percentage versus percentage of the sample population for the WATCH children

**Figure 2:**
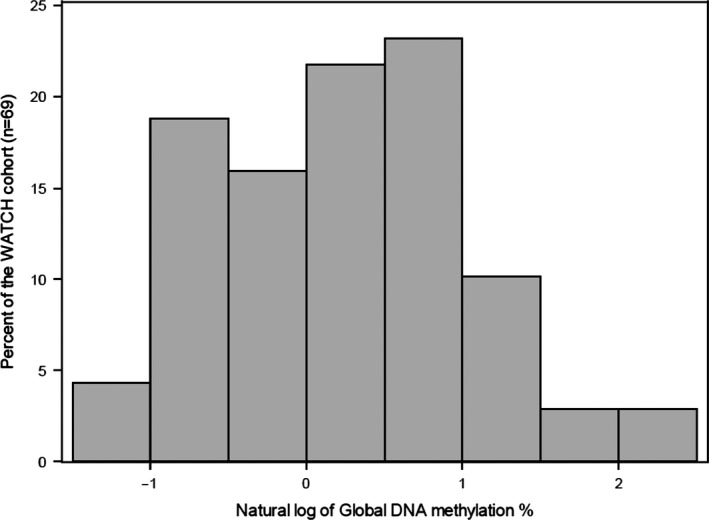
Frequency histogram for the natural logarithm transformation of global DNA methylation percentage versus percentage of the population for the WATCH children

The distribution of the global DNA methylation levels (%5mC) in buccal cells at 4 years of age was skewed to the right (Figure 1) with 93% of the samples between 0% and 3% methylated, the median was 1.32%, and the values ranged from 0.31% to 10.75%. Samples (7%) that were >3% methylated are considered outliers but were included due to the small size of the study. Global DNA methylation levels (%5mC) were significantly (*p* = .01) higher in males compared to the females [median (interquartile range (IQR)) 1.82(0.61–1.86) vs. 1.03(0.99–2.73)]. The global DNA methylation data percentage was log‐transformed to satisfy normality assumptions (Figure 2). The median(IQR) cognition scores at 4 years of age were full‐scale IQ 108(101–114), verbal IQ 103(98–111), performance IQ 107(100–118), PSQ 109(104–114), and GLC 108(97–116). The following behavioral scores could not be modeled due to the sparsity of their distributions: emotionally reactive 54(50–81), anxious/depressed 54(50–58), somatic complaints 50(50–62), withdrawn 73(54–84), sleep problems 54 (50–73), attention problems 54(50–69), aggressive behavior 50(50–69), and stress 62(54–85). The median(IQR) behavior scores at age 4 years for total problems, internalizing, and externalizing were 42(17–69), 46(21–73), and 38(19–73).

The linear regression models suggest that all cognition and behavioral scores decreased for each additional unit increase in log‐transformed global DNA methylation, though all effects were small and none were statistically significant (Table 2). All models were adjusted for age at completion of behavioral/cognition assessment. The R‐squared values for the linear regression models indicated that each adjusted model accounted for only 2% to 6% of the variation in the cognitive or behavioral outcomes.

**Table 2:** Association of buccal global DNA methylation with cognition and behavioral outcomes for the WATCH children

Model^a^	*N*	Variable^b^	Outcome	Association (95% CI)	*p*‐Value	*R* ^2^
1	56	DNA methylation %	Full‐scale IQ	−1.73 (−5.22 to 1.77)	.33	.02
2	57	DNA methylation %	Verbal IQ	−1.49 (−4.81 to 1.84)	.38	.02
3	57	DNA methylation %	Performance IQ	−0.859 (−5.02 to 3.31)	.69	.03
4	49	DNA methylation %	Processing speed composite	−1.847 (−4.99 to 1.29)	.25	.03
5	57	DNA methylation %	General language composite	−2.175 (−7.06 to 2.71)	.38	.02
6	56	DNA methylation %	Total problems score	−1.943 (−13.14 to 9.25)	.73	.06
7	56	DNA methylation %	Internalizing broad band score	−3.257 (−15.09 to 8.57)	.59	.05
8	56	DNA methylation %	Externalizing broad band score	−0.043 (−11.00 to 10.92)	.99	.05

^a^ All models were adjusted for age at cognition and behavioral assessment.

^b^ The natural logarithm transformation of global DNA methylation was used for the linear regression models to meet normality assumptions.

## 4 DISCUSSION

Examining whether associations exist between DNA methylation and cognitive and behavioral outcomes is important for understanding whether early epigenetic programming contributes to variation in child cognitive function and behavior. One‐carbon metabolism is regulated by dietary nutrients and is required for DNA methylation; therefore, this mechanism potentially contributes to the association between breastfeeding and child cognition. Emerging human studies suggest that breastfeeding is negatively associated with DNA methylation of specific loci including the leptin (LEP) gene, implicated in appetite regulation and fat metabolism (Obermann‐Borst et al., 2013), and the cyclin‐dependent kinase inhibitor 2A (CDKN2A) gene, involved in the production of tumor compressor proteins (Tao et al., 2013). However given the very limited evidence available, further investigation is warranted.

Previous studies have shown that prenatal DNA methylation patterns are associated with cognitive function and behavior during infancy and childhood (Lesseur et al., 2014; Lillycrop et al., 2015). To our knowledge, this is one of few studies that has explored associations between early childhood DNA methylation patterns with cognition and behavior. Therefore, the current study should act as a design template for the development of future studies. No association was found between global DNA methylation and child cognitive and behavioral outcomes, although the sample size was small, hence limiting the ability to detect the relationship as statistically significant. Based on the study's reported regression model, we estimate that a sample size of 230 children would be required to detect a statistical significant (*p* < .05) association between global DNA methylation and cognition and behavioral outcomes, assuming that the true effect is as large as what we observed. The current data could potentially be pooled with other datasets in future meta‐analysis to determine the impact of DNA methylation on child cognition and behavior. Furthermore, the impact of DNA methylation patterns on gene expression and brain function may not be apparent until later in life, which will require further exploration.

### 4.1 Child cognition and behavior

This study used WPPSI‐III and CBC, which are multiple domain based assessments for child cognition and behavior that are applicable to a broader research setting. However, there are methodological issues that impact on the accuracy of child cognition and behavior assessments (Isaacs & Oates, 2008). A child's performance during cognition and behavioral assessments can be affected by their mood, motivation, anxiety, energy levels, and personal effort. Environmental factors including noise, temperature, lighting, and who conducts the assessment can also influence child performance (Isaacs & Oates, 2008). However, preschool age (3–4 years) cognitive and behavioral assessments can differentiate between specific cognitive skills (e.g., verbal and spatial skills) and are likely to be more predictive of their future IQ than assessments in younger children (Volpe, 2017). The WATCH cohort reported similar full‐scale IQ scores to the SWS cohort that also measured cognition in healthy four‐year‐old children using the WPPSI. The SWS reported a median full‐scale IQ of 110, which is only two IQ points greater than the WATCH cohort (Lillycrop et al., 2015). Both these cohorts generally reported a high level of maternal education and low prevalence of maternal smoking which may have contributed to a full‐scale IQ that exceeded the mean of 100.

### 4.2 Gender differences in DNA methylation

Global DNA methylation was found to be higher in males compared to females, which is in agreement with previous studies (Boeke et al., 2012; Huen et al., 2014; Perng et al., 2012). Although the absolute gender difference in DNA methylation is small (0.79%) at a global level, this may be functionally significant at a locus‐specific level. Sexual differentiation of DNA methylation patterns occurs during implantation and embryo development. For example, in males, DNA methylation controls the timing of the sex‐determining region Y chromosome (Sry) gene expression which initiates the development of the testis (Nishino, Hattori, Tanaka, & Shiota, 2004). In females, one of the two X chromosomes is transcriptionally silenced by hypermethylation and chromatin modifications, which equalizes the dosage of genes between males and females (Avner & Heard, 2001).

Evidence also suggests that significant DNA methylation changes occur during the postnatal period (Gilsbach et al., 2014; Weaver et al., 2004). Gender‐specific differences in epigenetic programming could be attributed to the actions of steroid hormones, which are known to influence DNA methylation and gene expression, and thus modulate the differentiation of organs and tissues (Wood, Washburn, Mukherjee, & Banerjee, 1975; Gonzales, Ballard, Ertsey, & Williams, 1986). In males, a surge in the testicular hormone testosterone occurs at age 1–3 months, which has a role in the sexual dimorphism of the brain (Winter, Hughes, & Reyes, 1976). Testosterone is metabolized to estradiol or dihydrotestosterone, which can then act on estrogen and testosterone receptors in the brain (Gabory, Attig, & Junien, 2009). Evidence from animal models indicates that estradiol can alter DNA methylation patterns and estrogen receptor expression resulting in structural and behavioral sexual differentiation of the brain (Kudwa, Bodo, Gustafsson, & Rissman, 2005; Kurian, Olesen, & Auger, 2010; Westberry, Trout, & Wilson, 2010).

### 4.3 Locus‐specific DNA methylation

In the current study, there were no statistically significant correlations with measures of cognition and methylation at a global level detected in the WATCH cohort. However, significant changes in methylation at a locus‐specific level cannot be excluded. The SWS cohort identified 41 loci that at birth were associated with WPPSI full‐scale IQ at age 4 years (Lillycrop et al., 2015). More specifically, the SWS study found an association between hairy and enhancer of split‐1 (HES1) gene and WPPSI full‐scale IQ in children (*n = *175) at age 4 years (Lillycrop et al., 2015). These findings were also confirmed within the GUSTO cohort (*n = *108), demonstrating that HES1 DNA methylation was correlated with infant behavior at one year of age (Lillycrop et al., 2015). HES1 provides an important role in the Notch signaling pathway, which is necessary for neural development and differentiation (Richards & Rentzsch, 2015; Sestan, Artavanis‐Tsakonas, & Rakic, 1999). Recent animal studies have shown that Notch is required for memory consolidation (Dias et al., 2014; Yoon et al., 2012). In addition, Paquette et al. (2016) found a correlation between placental DNA methylation ankyrin repeat domain 11 (ANKRD11) genes and newborn attention. Deletions of the ANKRD11 gene are correlated with KBG syndrome (Ockeloen et al., 2015; Sirmaci et al., 2011), autism (Butler, Rafi, & Manzardo, 2015; Willemsen et al., 2010), and reduced nonverbal IQ (Willemsen et al., 2010). Based on this emerging evidence related to prenatal DNA methylation patterns, the association between locus‐specific postnatal DNA methylation patterns and cognition and behavior would be of interest in our cohort. Analyzing both global and locus‐specific DNA methylation is a comprehensive approach for understanding how specific DNA methylation patterns may influence gene expression and disease susceptibility.

### 4.4 DNA methylation surrogate tissue

DNA methylation patterns in live human brain tissue cannot be analyzed; therefore, the current study used buccal tissue as a surrogate. Buccal and neural cells may share similar DNA methylation patterns, as both cells originate from the ectoderm germ layer. Lowe et al. (2013) observed a 32% overlap in DMR DNA methylation (in regions <30% methylated) between buccal and brain tissue, when analyzed using bisulfite sequencing (BS‐seq) and reduced representation bisulfite sequencing (RRBS) data (Lowe et al., 2013). However, 75%–87% of the DMRs could not be captured by BS‐seq and RRBS; therefore, potential correlations and variations between the tissues may not have been detected (Lowe et al., 2013). Fisher et al. (2015) recently demonstrated that the CpG site cg23933044 was hypomethylated in buccal cells in twins (*n = *24) with reported psychotic symptoms at 12 years of age and in postmortem brain tissue of adults with schizophrenia (*n = *38; Fisher et al., 2015). However, recent evidence suggests that postmortem intervals and postsampling effects may induce changes in global DNA methylation levels in the brain; therefore, these findings should be interpreted with caution (Pidsley & Mill, 2011; Sjoholm, Ransome, Ekstrom, & Karlsson, 2018). To address the limitations of using postmortem brain tissue, Braun et al. (2019) compared genome‐wide DNA methylation in live brain tissue samples (*n* = 27) from patients aged 5–61 years with medically intractable epilepsy undergoing brain resection, with peripheral tissues (blood, saliva, and buccal). Findings from this study indicated that DNA methylation across subjects showed relatively high levels of cross‐tissue correlations (buccal–brain *r* = .85, blood–brain *r* = .86, saliva–brain *r* = .90; Braun et al., 2019). However, analysis of specific DNA CpGs and genes indicated that cross‐tissue correlations varied widely (Braun et al., 2019). The authors concluded that surrogate tissues for the brain may only be informative for a specific genomic region/s in which a high level of cross‐tissue correlation is demonstrated (Braun et al., 2019). As the sample size of the study by Braun et al. (Braun et al., 2019) was small, further research is needed to understand whether buccal cells can be used as surrogate tissue for the brain and in what context.

## 5 CONCLUSION

Due to limited statistical power, in the current study there is a high probability of a type 2 error; therefore, the lack of a demonstration of a correlation between global methylation of buccal DNA and measures of cognition should not be regarded as definitive. These results are valuable in identifying the size of required future studies to test the hypothesis, and these data could be pooled with that of other cohorts for meta‐analyses. The association between DNA methylation at a global and locus‐specific level, and cognition and behavioral outcomes in later childhood warrants further investigation. These data will be important for understanding whether DNA methylation is a potential mechanism that may explain the association between breastfeeding and child cognition.

## CONFLICT OF INTEREST

The authors declare no conflicts of interest associated with the research presented.

## AUTHORS' CONTRIBUTIONS

RMT, AJH, CEC, RS, TJE, and DM were involved in the research design. RMT, MWWB, DM, ECC, TS, TB, and JRA were involved in conducting and analyzing the global DNA methylation of the WATCH cohort samples. NB and KD were involved in conducting and analyzing the child cognition and behavior assessments. RMT and TJE performed the statistical analysis. RMT drafted the paper and was primary responsible for the final content. All authors provided constructive feedback and approved the final manuscript.

## Data Availability

Research data are not shared.
